# Rheological Characterization and Modeling of Thermally Unstable Poly(3-hydroxybutyrate-co-3-hydroxyvalerate) (PHBV)

**DOI:** 10.3390/polym13142294

**Published:** 2021-07-13

**Authors:** Silvia Lajewski, Annika Mauch, Kalman Geiger, Christian Bonten

**Affiliations:** Institut für Kunststofftechnik, University of Stuttgart, Pfaffenwaldring 32, 70569 Stuttgart, Germany; a_mauch@t-online.de (A.M.); kalman.geiger@ikt.uni-stuttgart.de (K.G.); christian.bonten@ikt.uni-stuttgart.de (C.B.)

**Keywords:** bioplastics, PHBV, rheological characterization, plastics processing

## Abstract

Presently, almost every industry uses conventional plastics. Its production from petroleum and extensive plastic pollution cause environmental problems. More sustainable alternatives to plastics include bioplastics such as poly(3-hydroxybutyrate-co-3-hydroxyvalerate) (PHBV), which is produced by bacteria and is biodegradable even in seawater. High temperature sensitivity as well as massive thermal degradation cause difficulties during the processing of PHBV. The aim of this work is to create a detailed rheological characterization and master curves to gain deeper knowledge about the material and its processing parameters. The rheological characterization was performed with frequency sweeps in the range of 0.1 rad/s to 628 rad/s and time sweeps over 300 s. Creating master curves at the reference temperature of 180 °C with the software IRIS delivers Carreau and Arrhenius parameters. These parameters allow for a calculation of the master curves for all other temperatures by means of the temperature shift factor. Moreover, the rheological measurements reveal a minimum rheological measurement temperature of 178 °C and a surprisingly high activation energy of 241.8 kJ/mol.

## 1. Introduction

Presently, plastics are used for every imaginable application. Because of the industry’s focus on the benefits of the processing and application of plastics, important aspects of plastics have been neglected. In addition to the problematic use of finite resources such as petroleum, masses of plastic waste and the creation of plastic patches in oceans produce a major environmental threat [1]. In order to solve these problems without losing the positive properties of conventional plastics, the industry is in search of better alternatives. A very up-to-date and detailed overview of current research trends in biobased and biodegradable polymers can be found in [2]. A promising biopolymer that can make a small but important contribution along the way in the fight against the ocean garbage patches is poly(3-hydroxybutyrate-co-3-hydroxyvalerate) (PHBV).

PHBV belongs to the group of polyhydroxyalkanoates (PHA) and is a copolymer of polyhydroxybutyrate (PHB) [3]. The linear molecular structure of PHBV consists of a linear carbonyl chain with randomly distributed methyl and ethyl side-chains [4]. PHBV is a biodegradable, biocompatible, hydrophobic, and non-toxic polymer [5,6]. It is produced, for instance, by the Gram-negative bacterium Ralstonia eutropha as an energy and carbon store. The production takes place under a lack of oxygen, nitrogen, and phosphor as well as a surplus of carbon [7]. To produce the characteristic side-chains of PHBV, propionic acid and valeric acid are added [8]. The brittle and stiff thermoplastic polymer shows viscoelastic properties [9]. In addition to the application of PHBV for films and packaging, there is also research on applications in medical technology. However, due to high production costs as well as its brittle and temperature-sensitive behavior, PHBV is not currently established in industry and PHA only accounted for 1.7% of the global production capacities of bioplastics in 2020 [10].

Depending on the composition, the melting point of PHBV varies between 50 °C and 180 °C [11]. Temperatures above the melting point cause unstable behavior of PHBV. Thus, the influence of high temperatures leads to a strong degradation of the polymer chains. The thermal degradation processes with α-deprotonation, β-elimination, and random chain scission proceed via several intermediates. At the end of thermal degradation, propylene, acetaldehyde, ketene, acetone, and carbon dioxide occur [12,13]. 

Viscosity curves are suitable tools for the analysis of the rheological properties of PHBV. These measurements are carried out in the molten state and can therefore accurately reflect the processing behavior of the plastics considered in the application. Even though PHBV has so far been used mainly in blends, the rheological characterization of the pure material is also important in order to obtain a statement of the expected material behavior. Only in this way can the pros and cons for the use of this biopolymer be assessed.

**Figure 1:**
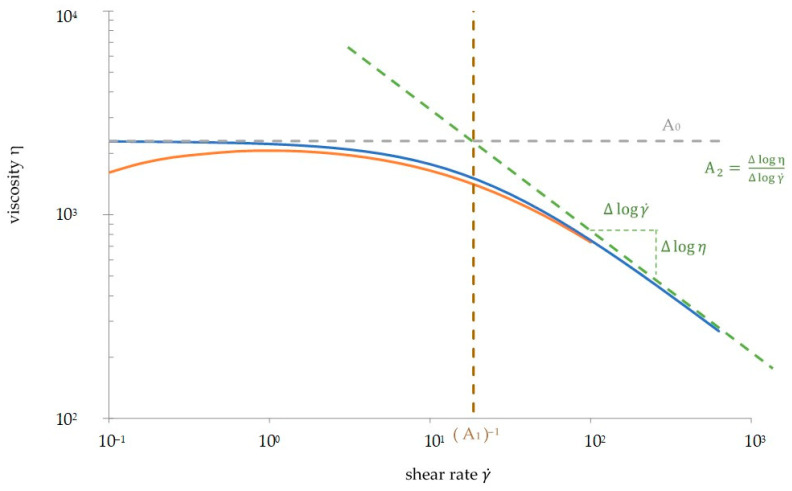
Viscosity curve of thermoplastics. The blue curve is an approximated viscosity curve derived from the Carreau model, and the orange curve is from the rheological measurement of PHBV.

The viscosity η is plotted double-logarithmically over the angular frequency ω or the shear rate $\dot{\gamma }$ (cf. Figure 1). A strong linear decrease at high frequencies is called the pseudo-plastic flow area or shear thinning behavior. At low frequencies, there is a constant viscosity called zero shear viscosity η_0_. Between the areas of zero shear viscosity and shear thinning, a transition occurs. The measured shear viscosity function of polymers with linear molecular structure (PS, PE-HD, PC, or even PHBV) is precisely approached with the Carreau model. The Carreau model, shown in Equation (1), calculates the viscosity η as a function of the shear rate $\dot{\gamma }$ with three parameters: the zero shear viscosity A_0_, the reciprocal transient shear rate A_1_, and the gradient of the pseudo-plastic flow area A_2_.

(1) $$\eta \left(\dot{\gamma }\right)=\frac{A_{0}}{{\left(1+A_{1}\dot{\gamma }\right)}^{A_{2}}}$$

Because of the temperature sensitivity of thermoplastics, the viscosity curve shifts with the change of temperature. At higher temperatures, the viscosity curve shifts by −45° [14,15]. According to Equation (2), the temperature shift can be calculated in the Carreau model (3) with the temperature shift factor a_T_:

(2) $$\eta \left(\dot{\gamma }\right)=\frac{a_{T}\cdot A_{0}}{{\left(1+a_{T}\cdot A_{1}\dot{\gamma }\right)}^{A_{2}}}$$

The nondimensional temperature shift factor a_T_ results from the Arrhenius model as the best approach for the temperature-dependent viscosity function of semi-crystalline polymers like PHBV and can be calculated following Equation (3) [15,16,17]:

(3) $$a_{T}=\exp\left(\frac{E_{0}}{R}\left(\frac{1}{T}-\frac{1}{T_{0}}\right)\;\right)$$

To plot viscosity curves, rheological measurements are necessary. Important values of a rheological measurement are the storage modulus G’ and the loss modulus G’’. G’ contains the reversible elastic energy, whereas G’’ represents the irreversible viscous energy component.

The complex viscosity can be used for an easier analysis of rheological measurements. As shown in Equation (4), the complex viscosity is calculated via the ratio of shear stress τ and the shear rate $\dot{\gamma }$ or the angular frequency ω [18].

(4) $$\eta^{*}=\frac{\tau \left(t\right)}{\dot{\gamma }\left(t\right)}=\frac{\tau_{0}\sin\left(\omega t+\delta \right)}{\gamma_{0}\omega \cos\left(\omega t\right)}$$

The empirical approach of Cox–Merz [19] in Equation (5) specifies the composition between the viscosity η and the amount of the complex viscosity η*. At low frequencies, the viscosity corresponds to the amount of the complex viscosity if the shear rate takes the same value as the angular frequency [16].

(5) $$\eta \left(\dot{\gamma }\right)=\left|\eta^{*}\left(\omega \right)\right|\;\mathrm{if}\;\dot{\gamma }=\omega$$

Thus, the graph of the complex viscosity and angular frequency can be linked with the graph of the viscosity and the shear rate.

In a preliminary work, viscosity curves of PHBV were plotted from rheological measurements. A frequency sweep (FS) from 100 rad/s to 0.1 rad/s was performed to obtain the viscosity curves. The viscosity curves did not fulfill the expected and discussed model curves. As shown in Figure 1, the viscosity decreases at frequencies lower than 10 rad/s where a constant zero shear viscosity is expected. This decrease can be explained with thermal degradation.

Due to the extreme thermal degradation already occurring just above the melting temperature, it is difficult to accurately predict viscosity curves at temperatures below or above the measurement temperature. Moreover, the reasonable range of temperature and frequency as well as the zero shear viscosity at a certain temperature are unknown. However, for further rheological measurements, applications, and processing of PHBV, it is necessary to gain this knowledge. Therefore, the aim of this work is to determine important rheological parameters of PHBV. Additionally, we try to better understand the thermal degradation of PHBV by comparing and analyzing measured and calculated viscosity curves.

## 2. Materials and Methods

### 2.1. Material

PHBV pellets from TianAn Biopolymer, Ningbo, China, of type ENMAT Y1000P were used for all measurements. According to the manufacturer, this type has a valerate content of only 1–2%. The molar mass was described in the literature as 485,000 g/mol [20]. The pellets were dried overnight in a vacuum drier from Binder GmbH, Tuttlingen, Germany, at 40 °C.

### 2.2. Methods

Rheological measurements were performed on the computer-controlled plate–plate rheometer Discovery HR-2 from TA Instruments, New Castle, Great Britain. A plate–plate rheometer measures torque with a rotatable upper plate. Using torque, angular frequency, and geometry data, the complex viscosity and angular frequency can be calculated [14,21]. Additional data from the rheological measurement are the storage and loss modulus.

At the beginning of the measurement, the pellets were placed at the lower plate and molded for three minutes. After three minutes, the gap of 1 mm was approached and the excess mass was removed. Three minutes later, the measurement was started as a frequency sweep or a time sweep. In the case of the frequency sweeps, the measurement was made from high to low frequencies. The parameters for the rheological measurement are presented in Table 1.

The software IRIS Rheo-Hub 2020 from IRIS Development LLC, Amherst, MA, USA was used to evaluate the measured data. The horizontal temperature shift is calculated with the “t-T-Shift mode” function. For the viscosity shift with the Carreau model, the function “Viscosity Fit -> 2nd Carreau” is used. All shifting parameters and data can be exported.

## 3. Results and Discussion

### 3.1. Preliminary Measurement for Error Analysis

**Figure 2:**
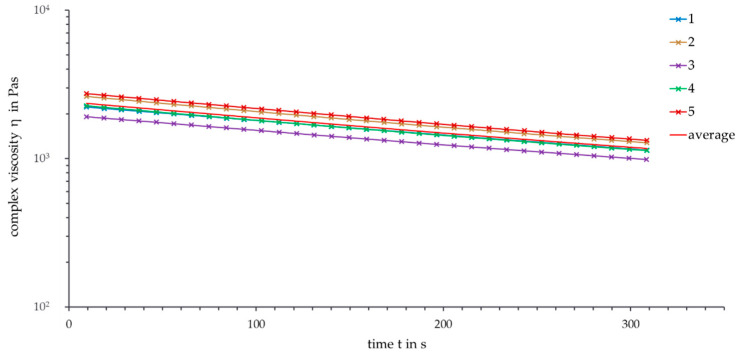
Error analysis, time sweep (ϑ = 180 °C, ω = 1 rad/s).

In order to analyze the fluctuations at several rheological measurements, a time sweep for 300 s at a temperature of 180 °C and a frequency of 1 rad/s was set five times. Figure 2 shows the semi-logarithmical plot of the complex viscosity over time.

High deviations between the measurements occurred, even though all measurements were set under the same conditions. At the beginning of the measurements, the standard deviation amounted to 12.55% and decreased to 10.27% after five minutes. The decrease of the standard deviation can be explained by the decreasing viscosity and longer measurement time. Further measurements at 190 °C show a lower standard deviation between 10.39% and 7.94%. Thus, the high standard deviation can be explained not only by the material properties of PHBV but also by the rheological measurement process, which is susceptible to inaccuracies in handling. For the following discussions and analysis, the standard deviation is given as 12.5%. 

### 3.2. Minimum Rheological Measurement Temperature

**Figure 3:**
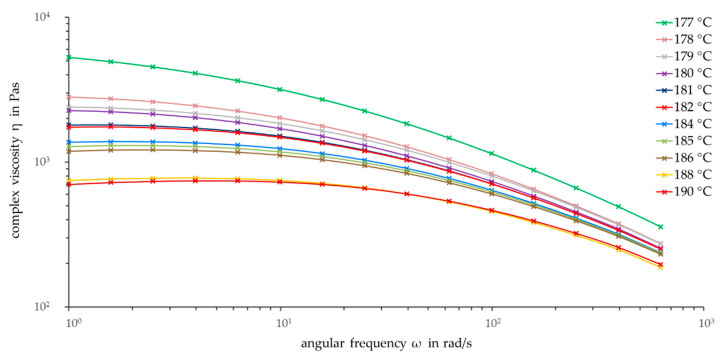
Frequency sweeps at different temperatures (ϑ = 177–190 °C, ω = 1–628 rad/s).

Thermal degradation has a huge impact on the viscosity curves of PHBV. Therefore, it is helpful to work with low measurement temperatures to minimize degradation effects. In order to investigate the impact of temperature on PHBV and to find the minimum measurement temperature, frequency sweeps were performed at different temperatures. Each frequency sweep at a certain temperature was measured three times, and the average was taken. For a clear illustration, error bars are omitted in Figure 3.

As can be seen, a higher measurement temperature resulted in a lower complex viscosity. Despite the high standard deviation, the viscosity curves are perfectly ordered according to temperature. The viscosity curves for 188 °C and 190 °C are visibly lower than the other curves. With the decrease in the complex viscosity between 10 rad/s and 1 rad/s, the thermal degradation of PHBV for high temperatures is illustrated. In the temperature range between 186 °C and 178 °C, the slopes of the viscosity curves above 100 rad/s are comparable. This can be explained by the assumption that in an ideal case the viscosity curves shift with the temperature by −45°. Consequently, the slope of −1, which is equal to a shift of −45°, shows a temperature invariance in the form of the viscosity curve. In Figure 3, the viscosity curves with different temperatures approach this temperature invariance with their behavior in the pseudo-plastic flow area. At lower frequencies, between 10 rad/s and 1 rad/s, the viscosity curves differentiate further. A zero shear viscosity has not yet been reached yet, especially at lower temperatures. 

The viscosity curve at 177 °C is remarkably higher than the curve at 178 °C. Due to the low temperature of 177 °C, the PHBV pellets cannot be completely melted with the given rheological measurement setup. Other measurements with longer melting times up to eight minutes showed the same effect. As is common with thermoplastic polymers, PHBV does not have a precise melting point due to the entangled polymer chains but rather a melting range where the polymer plastifies. Further research with differential scanning calorimetry (DSC) confirms this behavior: PHBV shows a pronounced melting peak with a peak temperature of 177.4 °C. Only complete melting of the sample material allows meaningful rheological measurements. Therefore, the minimum suitable temperature for rheological measurements of the PHBV pellets is 178 °C, which is an important parameter for its later processing.

It is worth mentioning that even a change in the measurement temperature of only 1 °C effects visible changes of the complex viscosity. This behavior indicates a high temperature dependency on the viscosity of PHBV and is a challenge in the processing and application of PHBV.

### 3.3. Master Curve at the Reference Temperature of 180 °C

With the measurement data from temperatures between 178 °C and 181 °C, it is possible to calculate a master curve for the reference temperature of 180 °C via IRIS. This master curve delivers the Carreau parameters A_0_ = 2297.3 Pas, A_1_ = 0.054 s, and A_2_ = 0.605, as well as the Arrhenius parameter E_0_/R = 29,077.3 K, which includes the activation energy E_0_ = 241,763.2 J/mol. This value is unusually high. In the literature, data of 25–80 kJ/mol can be found for polymer melts in general [15].

**Figure 4:**
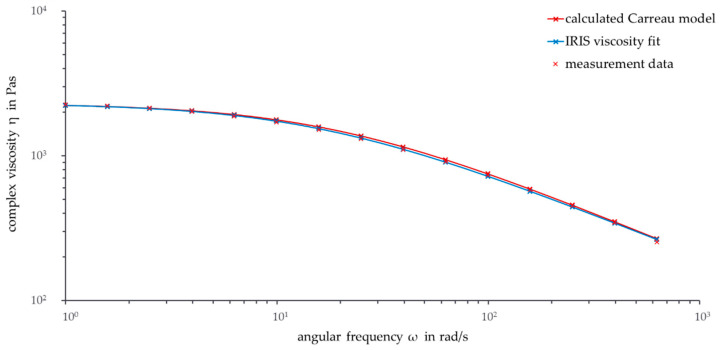
Frequency sweep: Calculated Carreau model, IRIS viscosity fit, and measurement data from reference temperature (ϑ = 180 °C, ω = 1–628 rad/s).

After calculating the temperature shift factors a_T_ with Equation (3), all master curves for other temperatures can be calculated by applying Equation (2). Figure 4 shows the calculated Carreau model as well as the IRIS viscosity fit and the measurement data for the reference temperature 180 °C.

The viscosity curve of the calculated Carreau model is similar to the IRIS viscosity fit. As the temperatures of the two curves correspond to each other, this behavior was expected. Between a frequency of 10 rad/s and 250 rad/s, the viscosity curve of the calculated Carreau model is slightly higher than the curve fitted with IRIS.

It is interesting to compare the calculated values at 180 °C with the values of a time sweep at 180 °C (not shown here). The Carreau model does not consider thermal degradation explicitly, so the calculated Carreau model values are considered ideal. Comparing the start data with the data of the time sweep after the same time as the frequency sweep underlines the temporal and thermal degradation of PHBV. These degradation processes intensify with increasing time and decreasing frequency. 

At a frequency of 100 rad/s, the calculated Carreau data are similar to the data of the time sweep after the same time. This could be an indicator that thermal degradation does not considerably affect the Carreau model because the master curve is based on data including thermal degradation. With decreasing frequency and increasing time, the conformity decreases due to increasing external influences. Especially for low frequencies, thermal degradation can be considered as having a minor impact on the calculated Carreau values.

Overall, the comparisons show that degradation processes occur at the reference temperature of 180 °C. The long preparation time of 6 min before a rheological measurement can start has to be considered. The discussed thermal degradation processes start before the start of the actual measurement. Actual processing steps, such as extrusion and injection molding, often need shorter times in the range of only a few minutes. For this reason, there should be deeper investigations into if and how the calculated data of the complex viscosity can be used for industrial processing.

### 3.4. Calculated Master Curves from the Parameters at the Reference Temperature 180 °C

In the following graphs, error bars for the calculated Carreau model curves are shown for easier analysis. The error bars are based on the above-mentioned standard deviation of 12.5% since the calculated Carreau parameters are based on measurement data including errors.

**Figure 5:**
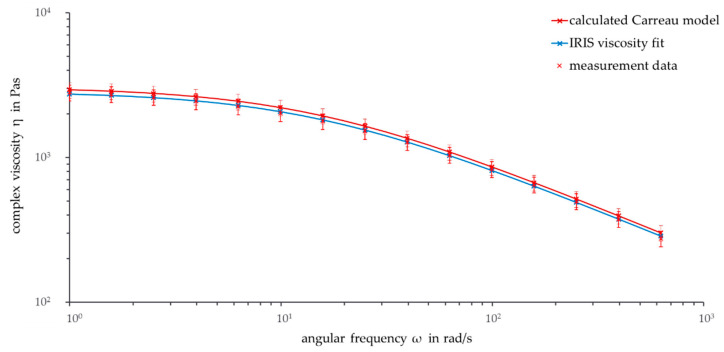
Frequency sweep: Calculated Carreau model, IRIS viscosity fit, and measurement data (ϑ = 178 °C, ω = 1–628 rad/s).

First, the viscosity curves from measurement temperatures below 180 °C are investigated. Figure 5 shows the double-logarithmically plotted complex viscosity over the frequency at the temperature of 178 °C. 

The calculated viscosity curve and the measured and fitted curve show a similar course, with the data of the calculated Carreau model curve being slightly higher than the measurement data. Compared to the data at 180 °C, the viscosity curves are shifted to higher viscosity values. This is due to the fact that the movement of the polymer chains slows down with a decrease in temperature, so the viscosity increases. As a result, the temperature shift factor increases for temperatures below the reference temperature.

If the measurement temperature exceeds the melting point, a stronger degradation of the PHBV chains occurs and results in lower complex viscosities. For temperatures above 182 °C, the complex viscosity curve of the calculated Carreau model is below the measurement data. It is notable that the difference between calculated and measured values increases with higher frequencies. This is interesting, because the deviation increases at lower frequencies for temperatures below 180 °C. The reason for increasing differences at higher frequencies can be found in the transition area of the viscosity curve. Especially at measurement temperatures above 185 °C, the superposition of degradation and viscosity change in the transition area begins after a shorter time and therefore at higher frequencies. Therefore, the transition area of the calculated Carreau model overlaps with a higher transition angular frequency. The Carreau model overestimates the temperature-dependent behavior of the complex viscosity at high temperatures. Higher values for the calculated viscosity were expected, because of the minor influence of degradation on the Carreau model.

**Figure 6:**
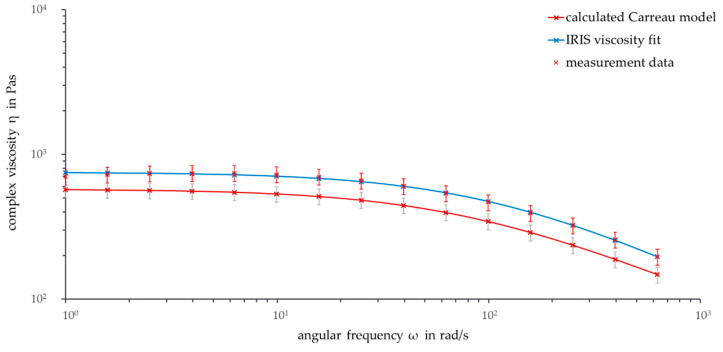
Frequency sweep: Calculated Carreau model, IRIS viscosity fit, and measurement data (ϑ = 190 °C, ω = 1–628 rad/s).

The viscosity curves at 190 °C presented in Figure 6 show further details. A temperature of 190 °C is the first temperature where no overlap within the standard deviations of calculated and measured data are found and where the viscosity curves can be clearly separated. A possible reason for this is that at 190 °C, the material reaches a state in which thermal degradation has a strong influence on the PHBV chains. In general, the Carreau model underestimates the viscosity curves at temperatures above the reference temperature. On the one hand, this is explainable, because a higher complex viscosity of the Carreau model without thermal degradation was expected. On the other hand, this could be a sign that thermal degradation of PHBV chains is not as high as assumed.

For temperatures below and near the reference temperature, the Carreau model overestimates the viscosity. All in all, the Carreau model calculates and illustrates the viscosity curve satisfyingly. Since the calculated viscosity curves up to a measurement temperature of 182 °C are higher than the measured data, an investigation into whether this is due to the proximity to the reference temperature or to the proximity to the melting temperature should be performed. 

**Figure 7:**
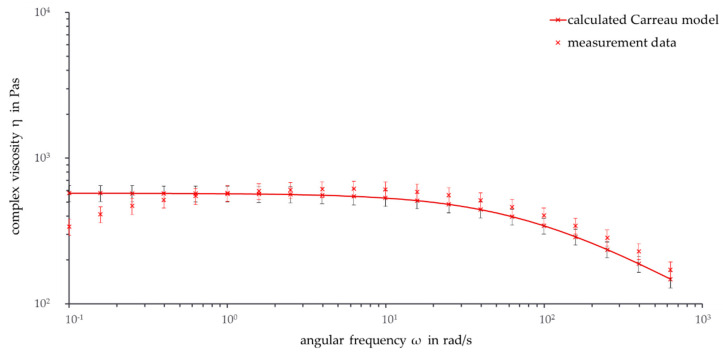
Frequency sweep: Calculated Carreau model and measurement data (ϑ = 190 °C, ω = 0.1–628 rad/s).

One possibility to further analyze the zero shear viscosity and the validity range of the viscosity curves is to extend the range of the curves from 0.1 rad/s to 628 rad/s. The analysis is only useful up to the frequencies where the calculated viscosity curve intersects with the measured viscosity curve (for high temperatures) or where the calculated viscosity curve deviates from the measured viscosity curves (for lower temperatures). Figure 7 shows the frequency sweep from 628 rad/s to 0.1 rad/s at 190 °C.

The measured viscosity curve crosses the calculated viscosity curve at 1 rad/s. For lower frequencies, the measured viscosity curve decreases while the calculated viscosity curve stays constant. Thus, the deviation between both viscosity curves increases with increasing angular frequency. At 0.25 rad/s, the error bars of the viscosity curves with the standard deviation intersect for the last time. As a result, the viscosity curve at 190 °C probably reaches zero shear viscosity between 1 rad/s and 0.25 rad/s.

The measured and calculated viscosity curves at a measurement temperature of 180 °C deviate with decreasing frequency. There is no intersection for the viscosity curves because the measured viscosity curve is lower than the calculated viscosity curve for the whole measurement. From 628 rad/s to 0.4 rad/s, there is an intersection of the error bars. Thus, the validity area at a measurement temperature of 180 °C starts at 0.4 rad/s.

## 4. Conclusions

The performed rheological measurements of PHBV are in the linear viscosity area and have a standard deviation of 12.5%. The minimum rheological measurement temperature of PHBV is 178 °C. Using the IRIS software, a master curve at the reference temperature of 180 °C based on the measurement data from 178 °C to 181 °C was calculated. This master curve delivers Carreau and Arrhenius parameters that can be used to calculate the master curves for other temperatures.

Master curves for measurement temperatures below the reference temperature show higher calculated values than the measurement data. At measurement temperatures above 182 °C, the calculated viscosity data are lower than the measurement data. Thus, the Carreau model underestimates the viscosity of PHBV at higher temperatures. The error bars of the master curves until 185 °C intersect with the error bars from the measurement data, suggesting that the master curves from 178 °C to 185 °C can be used for analysis. Due to the extension of the frequency area to 0.1 rad/s, the zero shear viscosity for 190 °C can be set at 1.0 rad/s to 576.5 Pas. Analogously, the zero shear viscosity for 180 °C can be assumed at 0.4 rad/s as 1732.7 Pas.

The investigated procedure to create master curves with the Carreau model results in viable curves. To minimize the difference between calculated and measured data, other calculation models should be investigated in the future. For example, the calculation of the temperature shift factor with the Vogel-Fulcher and the WLF models should be investigated in further projects. The molar mass also has a great influence on the rheological properties of PHBV. An analogous investigation of different PHBV types would provide further highly interesting insights into the degradation behavior of PHBV.

The rheological characterization underlines the high temperature sensitivity of PHBV. A temperature shift as small as 1 °C shows visible differences in the viscosity curves. This is further confirmed by the unusually high flow activation energy E_0_ = 241.8 kJ/mol and leaves only a small temperature window for processes and applications. More detailed investigations will allow better characterization of PHBV and allow for applications of the biodegradable plastic PHBV in the future.

## Figures and Tables

**Table 1:** Measurement parameters of the rheological measurements.

	Frequency Sweep	Time Sweep
preparation/molding time in min	6	6
plate diameter in mm	25	25
gap in mm	1	1
temperature in °C	177–190	180–190
angular frequency in rad/s	628.0–1.0 628.0–0.1	0.1 1.0 10.0 100.0
time in s	approx. 102	300
strain in %	5	5

## Data Availability

Not applicable.
